# Anthropometric status among infants with cleft lip and palate: a cross-sectional, single-center study in Indonesia

**DOI:** 10.1038/s41405-026-00468-3

**Published:** 2026-07-04

**Authors:** Andi Setiawan Budihardja, Patriana Yossy

**Affiliations:** 1 Dept/Oral maxillofacial Surgery, Faculty of Dentistry, Universitas Pelita Harapan Tangerang—Indonesia, and Comprehensive Cleft Center Siloam Lippo Village, Tangerang, Indonesia; 2 Faculty of Medicine, Universitas Pelita Harapan - Indonesia, Tangerang, Indonesia

**Keywords:** Paediatric dentistry, Health care, Dentistry

## Abstract

**Objective:**

Infants with cleft lip and/or palate (CL/P) may experience feeding difficulties that place them at increased risk of growth faltering. However, data comparing anthropometric outcomes between infants with CL/P and non-cleft infants in Indonesia remain limited. This study aimed to compare anthropometric indicators of undernutrition between infants with CL/P and infants without craniofacial anomalies evaluated at the same hospital.

**Methods:**

This cross-sectional study included 174 infants aged <1 year (87 with CL/P and 87 without craniofacial anomalies). Anthropometric data were obtained from electronic medical records at a single cleft center and the paediatric department of the same hospital. Length-for-age z-scores (LAZ) and weight-for-length z-scores (WLZ) were calculated according to World Health Organization (WHO) Child Growth Standards. Stunting was defined as LAZ <−2 standard deviations (SD), and wasting as WLZ <−2 SD. Associations between CL/P and anthropometric status were analysed using χ² tests.

**Results:**

The mean age was 6.0 ± 3.9 months in the CL/P group and 5.6 ± 2.6 months in the comparison group. Infants with CL/P had a significantly higher prevalence of stunting than infants without craniofacial anomalies (52.9% vs. 5.7%; *P* < 0.001). No significant difference was observed in wasting between the groups (2.3% vs. 2.3%; *P* = 0.069). Anthropometric status was not associated with cleft type (*P* = 0.989).

**Discussion:**

Infants with CL/P demonstrated a substantially higher prevalence of stunting, an indicator of chronic growth restriction, whereas wasting, an indicator of acute undernutrition, did not differ between groups. These findings suggest that growth deficits in infants with CL/P may accumulate gradually over time rather than manifest primarily as acute nutritional deterioration.

**Conclusion:**

Infants with CL/P may be vulnerable to chronic growth restriction during early life. Strengthening early feeding assessment, nutritional surveillance, caregiver education, breastfeeding support, and multidisciplinary cleft care may help reduce growth faltering and improve outcomes.

## Background

Cleft lip and/or palate (CL/P) is a congenital craniofacial abnormality that occurs during foetal development and is characterised by failed normal fusion of the palate and middle lip from the 5th to 12th week of gestation. CL/P is a common congenital anomaly with an estimated incidence of 1 in 700 live births [1, 2]. Its aetiology is multifactorial, with varying birth prevalence, including 0.3 per 1000 live births in African American populations, 1.0 per 1000 in Caucasians, 2.0 per 1000 in the Japanese population, and 3.6 per 1000 live births in Native North Americans [3, 4].

CL/P occurs in early pregnancy and affects a child’s nutritional status; children with CL/P are more prone to malnutrition [5]. This may be attributed to ineffective sucking due to inadequate pressure and regurgitation of milk into the nasal cavity. These problems result in infants with CL/P receiving lower nutrient intake than their normal counterparts. Many such babies die due to malnutrition before primary cleft lip repair can be achieved [6]. However, growth deficits observed among children with CL/P should not be considered an inevitable consequence of the congenital anomaly itself. Rather, nutritional outcomes are influenced by a complex interaction between feeding challenges, caregiver knowledge, availability of feeding support, healthcare access, socioeconomic conditions, and broader health-system factors. Poor nutritional status has also been associated with higher post-surgical complications, which hinder children with CL/P from receiving timely and safe surgical care [7, 8].

Several studies have reported an increased burden of undernutrition among children with CL/P. Delage et al. [9] analysed a global database of children undergoing primary cleft surgery and reported a higher prevalence of underweight compared with global estimates for children under five years of age. However, underweight represents a composite anthropometric indicator that does not distinguish between chronic growth restriction (stunting) and acute undernutrition (wasting). Other studies have evaluated anthropometric outcomes using different indicators and study designs, making direct comparison difficult.

In Indonesia, data regarding growth outcomes among infants with CL/P remain limited. Rafisa et al. [10] analysed nutritional status among children with CL/P across multiple Indonesian provinces and highlighted important regional variation in anthropometric outcomes. However, most available studies have not included a contemporaneous non-cleft comparison group evaluated within the same healthcare setting.

Therefore, the present study aimed to compare anthropometric indicators of undernutrition among infants with CL/P and infants without craniofacial anomalies who were assessed at the same hospital. We hypothesised that anthropometric status would differ between the two groups.

## Methods

### Ethical considerations

This study was approved by the ethics committee of our university (Approval No.: 072/K-LKJ/ETIK/II/2023). This manuscript was prepared and submitted in accordance with the STROBE (Strengthening the Reporting of Observational Studies in Epidemiology) reporting guidelines for cross-sectional studies. Informed consent was obtained from the children’s parents or guardians prior to data collection.

### Protocol and sample

This study was conducted at the comprehensive Cleft Center Siloam Hospital Lippo Village, Tangerang-Indonesia. The cleft center operates within the same hospital as the Department of Paediatrics from which the comparison groups was recruited. As part of routine multidisciplinary cleft care, caregivers received feeding advice and counselling from members of the cleft team. During the study period, specialised feeding appliances and presurgical infant orthopaedic devices were not routinely available for most patients because many families resided far from the treatment centre and were unable to attend frequent follow-up visits. Detailed information regarding feeding techniques, adherence to feeding recommendations, breastfeeding support received outside the hospital, and use of specialised feeding devices was not systematically recorded in the medical records and therefore could not be evaluated in this study.

The study population comprised infants aged <1 year and was divided into two groups: the CL/P group, which included infants with CL/P aged <1 year, and the control group, which included infants without CL/P aged <1 year.

The inclusion criteria for CL/P were age <1 year prior to any cleft surgery. The exclusion criteria for the CL/P group were as follows: (1) maternal alcohol consumption during pregnancy, (2) maternal smoking during pregnancy, (3) low birth weight (<2500 g), and (4) syndromic CL/P.

The number of participants (sample size) for this study was determined using an unpaired categorical comparative analysis formula. A total of 174 participants were included: 87 patients with CL/P aged <1 year and 87 infants aged <1 year. Data for the CL/P group were obtained from the preoperative electronic medical records of children who underwent primary cleft lip repair between 2017 and 2022 at a single Smile Train-sponsored center. Data for the non-CL/P group were obtained from children attending routine well-child visits and vaccination appointments and had no diagnosed craniofacial anomalies, chronic illnesses, or acute illnesses requiring hospitalisation

Weight and recumbent length measurements were obtained by trained healthcare personnel using standardised hospital procedures. Anthropometric status was assessed using World Health Organization (WHO) Child Growth Standards.

Length-for-age z-scores (LAZ) were used to assess stunting, an indicator of chronic growth restriction, whereas weight-for-length z-scores (WLZ) were used to assess wasting, an indicator of acute undernutrition. Z-scores were calculated using WHO Anthro software 3.2.2.

For analysis, children with LAZ < −2 standard deviations (SD) were classified as stunted and those with LAZ ≥ −2 SD as non-stunted. Children with WLZ < −2 SD were classified as wasted and those with WLZ ≥ −2 SD as non-wasted.

Because anthropometric measurements were obtained at a single time point, the study evaluated anthropometric status rather than longitudinal growth trajectories.

### Statistical analysis

Statistical analyses were performed using IBM SPSS Statistics for Windows, version 24 (IBM Corp., Armonk, NY, USA). The data were tested using the *χ*^2^ test, and *P*-values were assessed with continuity correction or Fisher’s exact test. Statistical significance was set at *P* < 0.05.

### Ethics approval and consent to participate

The study was approved by the ethics committee of our university (approval number: 072/K-LKJ/ETIK/II/2023). Participant were included in this study after obtaining oral and written informed consent.

## Results

Descriptive statistics for demographic data, including age and sex, in both the CL/P and non-cleft groups are presented in Table 1. The average ages of participants in the non-cleft and CL/P groups were 5.6 ± 2.6 months and 6 ± 3.9 months, respectively. The non-cleft group comprised 59.7% boys and 40.3% girls, whereas the CL/P group comprised 61% boys and 39% girls.

**Table 1:** Age and sex of the non-cleft and CL/P groups.

	Control		Study	
	*N* (%)	Mean $\pm \,$SD	*N* (%)	Mean $\pm \,$SD
Sex				
Male	52 (59.7)		53 (61)	
Female	35 (40.3)		34 (39)	
Age				
<6 months	37 (42.5)	5.6 $\pm \,$2.6	57 (65.5)	6 $\pm$ 3.9
≥6 months	50 (57.5)		30 (34.5)	
Total	87 (100)		87 (100)	

*CL/P* cleft lip and palate.

Unilateral cleft lip and palate (UCLP, 57.5%) was the predominant type of CL/P in this study population, followed by bilateral cleft lip and palate (BCLP) (26.4%), unilateral cleft lip (11.5%), bilateral cleft lip (2.3%), and cleft palate (CP) (2.3%) (Table 2).

**Table 2:** Types of defects in the CL/P group.

Type of defect	Total (*n*)	Percentage (%)
UCLP	50	57.5
BCLP	23	26.4
UCL	10	11.5
BCL	2	2.3
CP	2	2.3
Total	87	100

*CL/P* cleft lip and palate, *UCLP* unilateral cleft lip and palate, *BCLP* bilateral cleft lip and palate, *BCL* bilateral cleft lip, *CP* cleft palate.

According to the WHO length-for-age growth chart, 82 patients in the non-cleft group were non stunted (94.3%), and 5 were stunted(5.7%). In the CL/P group, 41 patients were non stunted (47.1%), whereas 46 were stunted (52.9%).

Based on the WHO weight-for-length growth chart, 85 patients in both the CL/P and non-cleft groups were non wasted, while 2 patients in each group were wasted.

The *χ*^2^ test was used to determine the significance of the relationship between the CL/P and nutritional status. Weight-for-length showed no significant relationship (*P* = 0.069, Fisher’s exact test) with CL/P, whereas length-for-age showed a significant relationship with CL/P (*P* < 0.001 with continuity correction). The results are presented in Tables 3 and 4.

**Table 3:** Chi-squared test results for the WHO length-for-age growth chart.

	Anthropometric category		Odds ratio	*P*-value
	Stunted (%)	Non stunted (%)		
CL/P	46 (52.9)	41 (47.1)	9.2	< 0.001
Non-CL/P	5 (5.7)	82 (94.3)		

OR = 18.40 [95% CI: 6.79–49.83].

*CL/P* cleft lip and palate, *WHO* World Health Organization, *OR* odds ratio, *CI* confidence interval.

**Table 4:** Chi-squared test results for the WHO weight-for-length growth chart.

	Anthropometric category		*P*-value
	Wasted (%)	Non wasted (%)	
CL/P	2 (2.3)	85 (97.7)	0.069
Non-CL/P	2 (2.3)	85 (97.7)	

OR = 1.00 [95% CI: 0.14–7.26].

*CL/P* cleft lip and palate, *WHO* World Health Organization, *OR* odds ratio, *CI* confidence interval.

Another *χ*^2^ test was conducted to determine the significance of the relationship between CL/P type and nutritional status, and found no significant relationship between unilateral and bilateral clefts and nutritional status (*P* = 0.989 with continuity correction), in addition to no significant relationship between cleft lip only and cleft lip with palate involvement and nutritional status (*P* = 0.296).

## Discussion

This study compared anthropometric indicators among infants with CL/P and infants without craniofacial anomalies evaluated at the same hospital in Indonesia. The principal finding was that infants with CL/P demonstrated a significantly higher prevalence of stunting, whereas no significant differences was observed in wasting. Previous studies have reported that children with CL/P have a higher risk of malnutrition than healthy children [8–10]. However, the observed association between CL/P and stunting should not be interpreted as evidence that the congenital anomaly itself directly causes undernutrition. Rather, growth restriction in this population likely reflects the cumulative effect of feeding difficulties interacting with caregiver support, access to healthcare services, feeding counselling, socioeconomic conditions, and broader health-system factors. These influences may begin shortly after birth and contribute to chronic growth deficits over time.

In this study, the average age of the patients was 5–6 months, and the sex distribution was similar between the CL/P and control groups. Malnutrition bore a significant association with CL/P based on the WHO length-for-age growth chart (representing long-term nutritional deficiency) (*P* < 0.001), as shown by the *χ*^2^ test; however, no significant results were observed between the WHO weight-for-length (showing short-term nutritional deficiency) growth chart and CL/P (*P* = 0.069). Moreover, the CL/P was associated with nutritional status, indicating a higher prevalence of malnutrition among infants with CL/P. The association between CL/P and long-term nutritional deficiencies in children appears to be significant. However, no short-term relationship was observed. Length for age and weight for length were selected to capture different dimensions of nutritional status and the different results yielded by each chart are therefore expected and informative. A length-for-age chart was used to examine the long-term relationship between CL/P and malnutrition in infants. Weight for length represents the association between infant malnutrition and CL/P over a short period.

The results of this study are similar to those of a previous study that reported a higher prevalence of malnutrition in infants with CL/P before cheiloplasty [11]. Although the WHO recommends exclusive breastfeeding for all infants aged <6 months, many infants with CL/P experience difficulty in breastfeeding [5, 12]. None of the infants in this study underwent any pre-surgical infant orthopaedics (PSIO) (e.g., a feeding plate) because they resided far from the hospital and could not make frequent visits for PSIO fabrication and check-ups. This may have contributed to the higher prevalence of malnutrition in the infants in the CL/P group.

In this study, malnutrition was not found to be associated with cleft type. This result differs from that of a previous study that concluded that the frequency of malnutrition was higher among infants with UCL/P than among those with BCLP. They hypothesised that the more severe facial disfigurement in BCLP forced parents to seek medical support earlier than in children with unilateral CL/P [13]. These results differ from those of a previous study [14] that reported that infants with CL/P were more prone to malnutrition than those with cleft lip. However, the underlying cause of this imbalance requires further investigation.

Previous studies have shown that exclusive breastfeeding is crucial for all infants; infants with CL/P can still breastfeed using special techniques and positions. For example, assisted feeding for infants with CL/P is recommended and reliable [15]. Numerous feeding techniques for these infants, such as the use of compressible bottles, modified nipples, cups, and feeding appliances, have been previously described [12]. Skilled feeding assistance to the mothers of affected infants is vital for lowering the risk of poor feeding and malnutrition, and to ensure timely and safe cleft surgery [16].

Tungotyo et al. [13] conducted a study in Uganda involving 44 infants with CL/P and reported that 68% were generally malnourished, with the highest burden among girls (71.4%), infants <4 months of age (73.5%), and infants with CL/P (77%). The lack of feeding provision and nutritional information for the caregiver was the major contributing factor to this result. Moreover, the Tungotyo study also assessed parents’ and caregivers’ knowledge of infant feeding practices for infants with CL/P. This knowledge can affect the nutritional status of infants. Nutrition educational programmes that integrate maternal education through counselling sessions, workshops, and other modalities may increase knowledge and skills related to infants’ feeding practices [17].

Several limitations should be considered when interpreting these findings. First, this was a single-centre study, which may limit generalisability to other populations. Second, the cross-sectional design precludes conclusions regarding causality and does not allow assessment of longitudinal growth trajectories. Third, information regarding feeding practices, breastfeeding success, use of specialised feeding devices, socioeconomic status, caregiver education, and household characteristics was not available. Fourth, anthropometric measurements were obtained at a single time point and therefore represent anthropometric status rather than growth over time. Future multicentre prospective studies incorporating detailed feeding and socioeconomic data are warranted. Future multicenter studies in cleft care facilities that consider feeding techniques, socioeconomic status, and parental education are necessary.

## Conclusions

Infants with CL/P in this study exhibited a significantly higher prevalence of stunting compared with infants without craniofacial anomalies, whereas no significant difference was observed in wasting. These findings suggest that infants with CL/P may be vulnerable to chronic growth restriction during early life. Growth deficits in this population are likely influenced by multiple interacting factors, including feeding difficulties, caregiver support, access to feeding counselling, healthcare access, and broader social determinants of health. This study highlights the importance of nutritional status in infants with CL/P, particularly in Indonesia. Our results align with those of previous studies that reported a high prevalence of malnutrition in children with CL/P. This finding is consistent with the commonly reported difficulties that infants with CL/P experience with breastfeeding and bottle-feeding. Most patients enroled in the study did not have any PSIO devices or feeding plates because of logistical issues related to the distance to the hospital; therefore, most patients were examined before cheiloplasty.

The WHO recommends exclusive breastfeeding for all infants aged <6 months, including those with CL/P. Achieving this can pose a major challenge for parents and healthcare providers. Thus, appropriate education and awareness regarding suitable feeding techniques should be disseminated to the parents of infants with CL/P. Additionally, such parents would greatly benefit from mandatory nutritional counselling. Moreover, public awareness of malnutrition in children with CL/P should be increased, and breastfeeding or feeding specialists should be included in cleft teams to assist mothers early after childbirth.
